# Glycogen Supplementation in Vitro Promotes pH Decline in Dark-Cutting Beef by Reverting Muscle’s Metabolome toward a Normal Postmortem Muscle State

**DOI:** 10.1021/acs.jafc.4c06490

**Published:** 2024-11-04

**Authors:** Frank Kiyimba, Steven D. Hartson, Gretchen G. Mafi, Ranjith Ramanathan

**Affiliations:** † Department of Animal and Food Sciences, Oklahoma State University, Stillwater, Oklahoma 74078, United States; ‡ Department of Biochemistry and Molecular Biology, Oklahoma State University, Stillwater, Oklahoma 74078, United States

**Keywords:** dark-cutting beef, glycogen, pH decline, metabolomics, substrate utilization

## Abstract

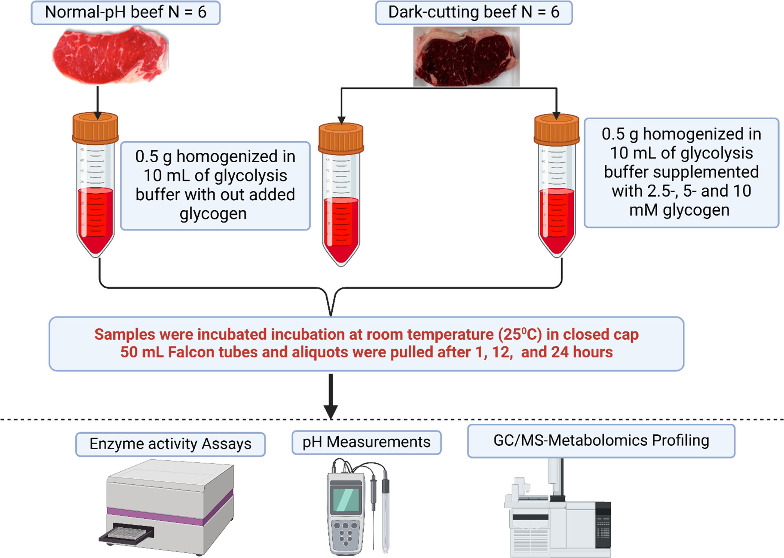

Dysregulated muscle glycogen metabolism preslaughter contributes to aberrant postmortem muscle pH (>5.8) in dark-cutting beef phenotypes. However, the underlying mechanisms have remained elusive. Herein, we examine the glycogen dependent regulation of postmortem muscle pH decline and darkening in beef. We show that supplementation of glycogen in vitro restores postmortem pH decline in dark-cutting beef by reverting the metabolome toward a typical postmortem muscle state characterized by increased activities of enzymes glycogen phosphorylase and lactate dehydrogenase (*p* < 0.05) coupled with a pronounced abundance of glycolytic metabolites and reduced abundance of tricarboxylic acid cycle and amino acid metabolites. Furthermore, concurrent inhibition of mitochondrial respiration at complexes I, IV, and V with glycogen supplementation stimulates greater pH decline. Together, our findings show that supplementing glycogen at low concentrations (10 mM) can reprogram the dark-cutting beef muscle’s metabolome toward typical postmortem state and promote muscle acidification. Thus, enhancing glycogen levels could represent a promising strategy for mitigating dark-cutting beef phenotypes and improving meat quality.

## Introduction

The rate and extent of postmortem pH decline play a pivotal role in determining meat color. The consumer-preferred bright cherry-red color is typically achieved at a postmortem muscle pH of approximately 5.6.^1−5^ Deviations from this norm, notably exceeding 5.8, are implicated in muscle darkening, a phenomenon extensively reported in several dark-cutting beef studies.^6−12^ The insights derived from these investigations highlight several biochemical implications: (i) defective postmortem muscle pH decline may promote enhanced mitochondrial respiration postmortem, elevating muscle oxygen consumption while diminishing oxygen availability for myoglobin binding, thereby increasing deoxymyoglobin levels, and (ii) elevated postmortem muscle pH can induce alterations in myofibril shrinkage, impacting the water content within the muscle, thus compromising its ability to reflect light, resulting in a darker meat appearance. While considerable knowledge has been acquired, significant gaps persist in our understanding of the molecular mechanisms and biochemical foundation underlying the pH-dependent regulation of muscle darkening in beef.

Muscle darkening is closely associated with depleted muscle glycogen and impaired glycogen metabolism preslaughter.^8,10,13^ During postmortem, stored muscle glycogen is mobilized and degraded into glucose, which then undergoes glycolysis to produce pyruvate. This pyruvate can either be transported into the mitochondria for oxidation via pyruvate dehydrogenase (PDH) catalyzed reactions or converted to lactate via lactate dehydrogenase (LDH).^14−18^ Previously, we demonstrated that dark-cutting beef muscles exhibit decreased abundance of glycogen breakdown enzymes (e.g., glycogen phosphorylase, bis-phosphoglycerate mutase, amylo-α-1-6-glucosidase, and phosphorylase b kinases) and several glycolytic metabolites (e.g., glucose, glucose-6-phosphate, fructose-6-phosphate), coupled with an overabundance of mitochondrial oxidative enzymes and metabolites.^6,10,12^ This metabolic alteration significantly impacts substrate metabolism in dark-cutting beef muscles, ultimately leading to compromised glycolytic flux and accumulation of lactic acid, which further contributes to dysregulated postmortem muscle pH decline. Based on this understanding, the amount of glycogen at slaughter regulates muscle acidification, in part, through production of lactate.^19−22^ However, other pathways involved in postmortem muscle pH decline^23,24^ could also be involved in dark-cutting beef.

The efforts to restore typical postmortem metabolic programs in dark-cutting beef muscles have explored various postharvest strategies, including the addition of specific metabolic substrate intermediates such as lactate enhancement^9,25^ and glucono-δ-lactone.^26^ While these approaches have shown signs of efficacy in improving both fresh and cooked meat color characteristics, alternative postharvest strategies, for example, utilizing an in vitro excess glycogen supplementation system (at 30 mM) in pork,^27^ lamb, chicken, and turkey,^22^ failed to rescue muscle pH decline in oxidative muscles. Additionally, feeding high starch diets as a preharvest strategy failed to impact glycogen content in slaughtered pigs^28^ and in cattle.^29^ In this study, we delve deeper into this issue by examining the potential of in vitro glycogen supplementation at low levels (2.5, 5, and 10 mM) to restore pH decline, enzyme activities, and postmortem metabolic programs, specifically in dark-cutting beef muscles, by characterizing the substrate metabolome postglycogen supplementation. We propose that inherent substrate inhibition mechanisms within dark-cutting beef disrupt normal glycogen metabolic programs, triggering downstream alterations in postmortem muscle metabolism, ultimately resulting in an elevated muscle pH phenotype.

## Materials and Methods

### Samples Collection and Preparation

Longissimus lumborum muscles from six bright-red normal-pH (Institutional Meat Purchasing Specification no. 180, NAMP, 2002) and six dark-cutting beef loins from A maturity (grain-finished, spray chilled) carcasses were procured from a local facility three days postmortem from Creek Stone farms, Arkansas City, KS. Vacuum-packaged loins were transported on ice to the Oklahoma State University Food and Agricultural Product Center. The loins were stored overnight in a cooler at 2 °C and fabricated on day four postmortem into 2.54 cm thick steaks. The first two steaks were used to measure color and muscle pH. The third steak was powdered in liquid nitrogen and used for subsequent glycogen addition analyses.

### Muscle Biochemical Analyses

Muscle color attributes and pH were determined following a previously published method.^30^ Briefly, muscle color characteristics (L*-, a*-, and b*-values) were determined by using a HunterLab MiniScan XE Plus spectrophotometer (HunterLab Associates, Reston, VA) with a 2.5 cm diameter aperture, illuminant A, and 10° standard observer. Muscle pH was determined using an Accumet 50 pH meter (Fisher Scientific, Fairlawn, NJ). The pH meter was calibrated with standard buffers at pH 4.0 and 7.0 and inserted into the meat at three different locations. The average pH of the steaks was measured and recorded.

### In Vitro Anaerobic Glycolysis

To simulate muscle glycolysis, 0.5 g of powdered longissimus lumborum muscle (*n* = 6 dark-cutting and *n* = 6 normal-pH beef) samples were homogenized and incubated in 10 mL of glycolytic buffer containing 10 mM Na_2_HPO_4_, 5 mM MgCl_2_, 60 mM KCl, 5 mM ATP, 0.5 mM ADP, 0.5 mM NAD+, 25 mM carnosine, 30 mM creatine, and 10 mM sodium acetate (pH 7.4),^27,31^ with or without glycogen in a 50 mL conical nonpyrogenic sterile centrifuge Falcon tubes (Corning, Glendale, AZ). pH 7.4 represents the physiological pH before animal harvest. Glycogen was supplemented at 0, 2.5, 5, and 10 mM. The control groups consisted of normal-pH and dark-cutting beef without added glycogen. Glycogen treated and controlled samples were incubated at room temperature (25 °C). Changes in pH, myoglobin redox state, metabolite profiles, and enzyme activities were monitored at 1, 12, and 24 h of incubation.

### pH

Aliquots were collected after incubation to determine the effect of glycogen supplementation on pH. Four volumes of homogenates at each incubation time point were added to one volume of buffer (25 mM sodium iodoacetate, 750 mM KCl, pH 7.0).^27^ Subsequently, the samples were centrifuged at 13 000*g* for 5 min, and the pH was measured immediately using an Acument 50 pH meter (Fisher Scientific, Fairlawn, NJ).

### Determination of Myoglobin Redox State

To evaluate the impact of glycogen supplementation on myoglobin redox state, the homogenate was centrifuged at 10 000*g* for 3 min. The resultant supernatant was used to determine myoglobin redox stability utilizing a spectrophotometric technique, scanning absorption from 400 to 700 nm with a microplate reader (Spectra Max M3, Molecular Devices, San Jose, CA). Myoglobin levels after 24 h of incubation in glycolysis buffer were determined for each treatment group (*n* = 6 each) by measuring wavelength maxima at 503, 525, 557, and 582 nm as per previous protocols.^32,33^ For this analysis, dark-cutting samples treated with 10 mM glycogen, which demonstrated the greatest pH decline, and control samples (normal beef and dark-cutting beef controls without added glycogen) were used.

### Analysis of Enzyme Activities

The activities of glycogen phosphorylase, lactate dehydrogenase, adenosine monophosphate activated protein kinase (AMPK), and phosphorylated AMPK (pAMPK) were measured using standard kits obtained from Abcam (Boston, MA). Aliquots of sample homogenates from controls (normal-pH and dark-cutting beef without added glycogen) and treated samples (dark-cutting beef supplemented with 10 mM glycogen) at 1 and 24 h of incubation were utilized for this analysis. Absorbance values for each enzyme activity were determined using a microplate reader (Spectra Max M3, Molecular Devices, San Jose, CA) following the protocols provided with the enzyme assay kits.

### Inhibition of Mitochondrial Oxidative Capacity

In a separate experiment, the influence of mitochondrial oxidative capacity on muscle glycolysis and the pH decline was investigated. Only samples treated with 10 mM glycogen, which demonstrated the greatest pH decline (Figure 2), were utilized in this experiment. Mitochondrial inhibitors targeting complex I (2 μM rotenone), complex IV (1 mM potassium cyanide), and complex V (2 μM oligomycin) were introduced into the glycolysis buffer containing powdered longissimus lumborum dark-cutting beef muscle supplemented with 10 mM glycogen. Control groups were maintained without the addition of inhibitors. Subsequently, tubes were gently inverted to mix and then incubated at 25 °C. Aliquots were removed after 1, 12, and 24 h of incubation and used to measure muscle pH decline and kept for metabolomics analysis.

### Global Metabolite Profiling

Metabolite profiling was conducted at the West Metabolomics Core Center, University of California, Davis. Metabolites were extracted from aliquots removed from the homogenates of powdered longissimus lumborum dark-cutting beef muscle supplemented with 10 mM glycogen (*n* = 6) and from dark-cutting and normal-pH beef without added glycogen (*n* = 6) after 1 and 24 h of incubation. Additionally, aliquots from dark-cutting homogenates supplemented with both 10 mM glycogen and mitochondrial inhibitors at complexes I, IV, and V were included in the analysis. Metabolite profiling was performed as described previously.^6^ Detail discussed by Fiehnn et al.,^34^ such as injector, column, and mass spectrometric settings, were utilized for various steps in the metabolomics profiling. Quality control was performed before each batch of 10 runs. The list of metabolites features quantified and identified in all treatment groups after 1 and 24 h of incubation were analyzed for differential abundance using MetaboAnalyst (V.6.0, https://www.metaboanalyst.ca).

### Statistical and Bioinformatics Analysis

A completely randomized design with a factorial arrangement was used to evaluate the combined effects of glycogen supplementation on muscle pH decline, myoglobin redox stability, and enzyme activities. The fixed effects/factors included glycogen, muscle type (dark-cutting vs normal-pH beef), incubation time, and their interactions. Overall, the experiment was replicated 6 times (*n* = 6). The enzyme activity experiment was replicated 5 times (*n* = 5) for glycogen phosphorylase and lactate dehydrogenase, while the AMPK and pAMPK activity study was replicated 3 times (*n* = 3). The data were analyzed using the Mixed Procedure of SAS (version 9.4, SAS Inst. Inc., Cary, NC). Least square means were separated using a pairwise *t* test and were considered significant at α = 0.05. Exact *p*-values are indicated, and each dot in a bar graph represents a biological replication, with error bars in the figure representing the standard error of the mean.

The metabolite data sets were normalized by a median, log-transformation, and scaled by Pareto scaling using MetaboAnalyst (V.6.0, https://www.metaboanalyst.ca). One-way analysis of variance was implemented to identify differentially abundant metabolites across all treatment groups at 1 and 24 h of incubation with/without glycogen supplementing using Tukey LSD. Furthermore, pairwise comparison was conducted by comparing glycogen treated samples against control groups. Metabolites with a false discovery rate (FDR)-adjusted *p* value <0.05 and a Log2-fold change (FC) >2 or less than −2 were considered differentially abundant. Principal component analysis (PCA), supervised projections to latent structure-discriminant analysis (PLSDA) on the first two of 10 components, and hierarchical cluster analysis were performed to create plots and heat maps for the differentially abundant metabolites. Venn diagrams were constructed to visualize the distribution of the differentially abundant metabolites using jven^35^ (https://jven.toulouse.inra.fr/).

### Biochemical Pathway Analysis

Pathway enrichment analysis was performed on metabolites with fold expression ratio of ≥±2 at a false discovery rate (FDR)-adjusted *p* value >0.05 for specific treatment comparisons was conducted using MetaboAnalyst (V.6.0, http://www.metaboanalyst.ca) with reference to the KEGG pathway database for *Bos taurus*. Furthermore, over-representation analysis was performed using the Fisher’s extract test and pathway topology analysis using relative betweenness centrality.

## Results

### Dark-Cutting Beef Muscle Color Characteristics and Biochemical Profiles

**Figure 1:**
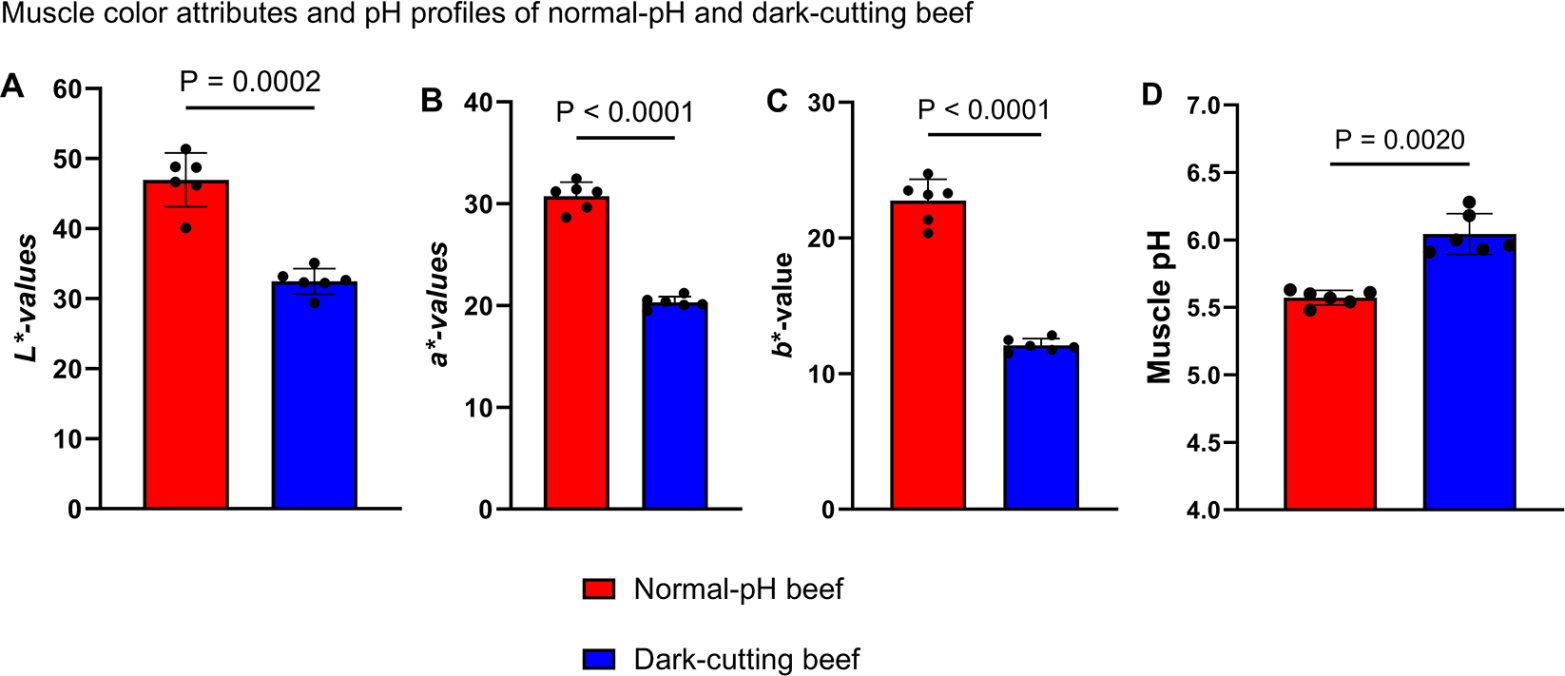
Comparison of muscle surface color characteristics and pH between dark-cutting and normal beef. (A–C) Statistical summary reveals lower levels of lightness (L*), redness (a*), and yellowness (b*) in dark-cutting beef compared with normal beef (*n* = 6 per group, *t* test, *P* < 0.01). (D) Dark-cutting beef exhibits a significant increase in muscle pH relative to normal beef (*n* = 6 per group, *t* test, *P* < 0.01). The error bars represent ± standard error of mean (SEM) with *p* values; each dot in a bar graph represents a biological replicate.

As expected, dark-cutting beef muscles used in the current study showed lower L*, a*, and b* values compared with normal-pH beef (Figure 1A–C; *p* < 0.05). Our results are consistent with previous reports that found lower values in dark-cutting beef compared to normal-pH beef. The lower L* values represent a low muscle lightness, while lower a* values show that dark-cut beef has a lower red intensity. Further assessment of muscle pH also confirmed significantly greater pH values in the dark-cutting beef longissimus lumborum muscles compared with normal beef muscles (Figure 1D; *p* < 0.05). Together, these results demonstrate that dark-cutting muscles used in the current study met the benchmark characteristics of dark-cutting phenotypes reported in beef.^6,10,12^

### In Vitro Glycogen Supplementation Promotes Postmortem pH Decline in Dark-Cutting Beef Muscle via Substrate-Mediated Activation of Enzymes Involved in Glycogen Metabolism

**Figure 2:**
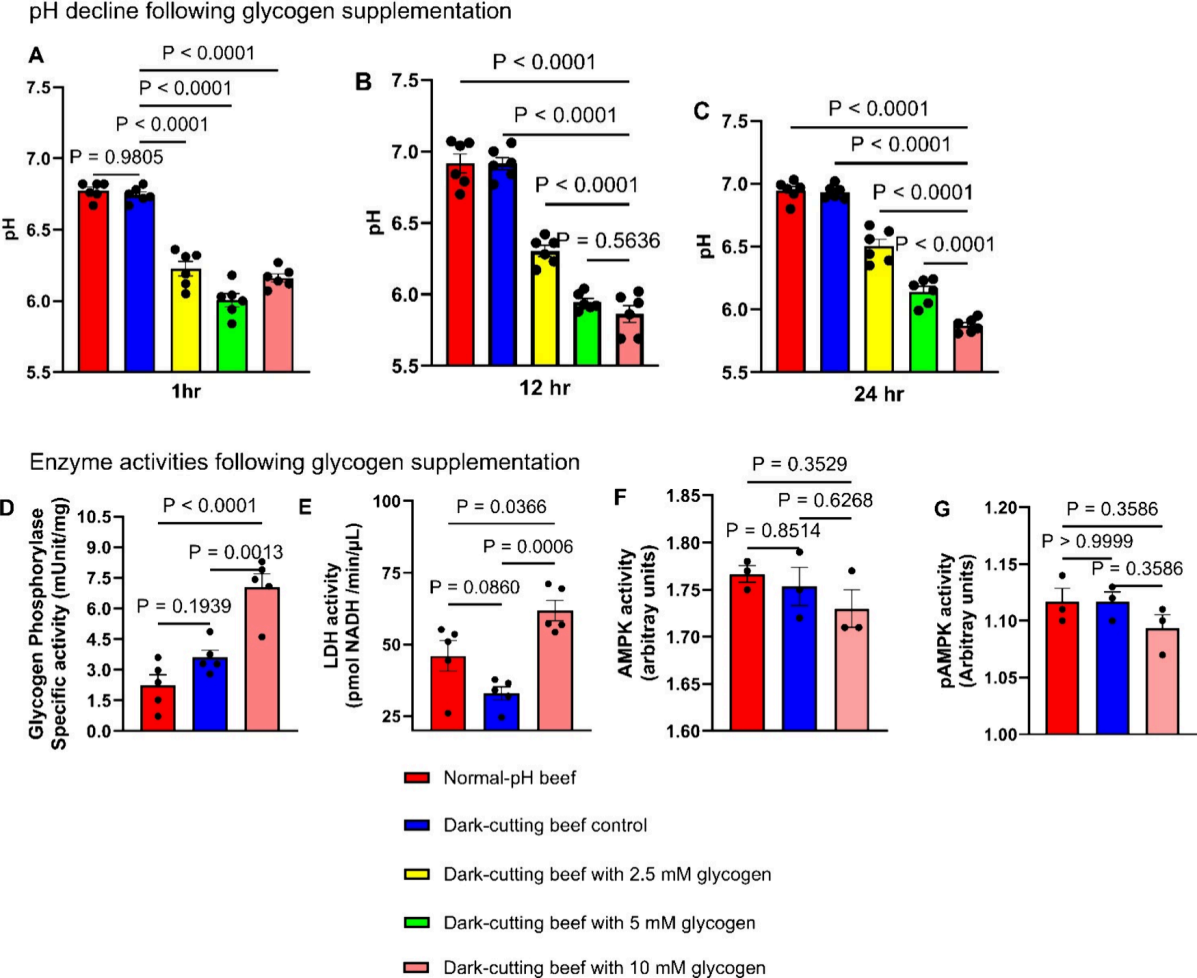
Glycogen supplementation facilitates muscle pH decline and enhances the activity of glycogen degradation and lactate formation enzymes in dark-cutting beef. (A–C) Summary of pH changes in response to glycogen supplementation at concentrations of 2.5, 5.0, and 10 mM after 1, 12, and 24 h of incubation, respectively. (D–G) Enzyme activities of glycogen phosphorylase (D), lactate dehydrogenase (E) (*n* = 5 per group, ANOVA, *p* < 0.001), AMPK (F), and pAMPK (G), measured after 24 h of incubation (*n* = 3 per group, ANOVA, *P* > 0.001). Error bars represent ± standard error of the mean (SEM) and *p* values; each dot in a bar graph represents a biological replication.

The abnormal postmortem muscle pH in dark-cutting beef muscles might indicate a distal bottleneck in glycogen metabolic programs and/or inherent substrate inhibition mechanisms. To evaluate this possibility, we supplemented dark-cutting beef muscle homogenates with different glycogen levels (0, 2.5, 5, and 10 mM) using an in vitro glycolysis system (Figure 2). Results showed that glycogen supplementation at 2.5, 5, and 10 mM significantly reduced pH after 1 h, with the 5 mM group exhibiting the most pronounced decline (Figure 2A). After 24 h of incubation, the 10 mM group showed the greatest pH decrease (*p* < 0.05; Figure 2C). This suggests that extended exposure to higher glycogen concentrations may lead to the accumulation of metabolic intermediates, driving a more substantial decline in pH. These changes in pH following glycogen supplementation could, in part, be attributed to alterations in substrate utilization and mobilization.

To confirm this possibility, we assessed the activities of the enzymes glycogen phosphorylase (PYGM) and lactate dehydrogenase (LDH), which are associated with glycogen breakdown and muscle acidification, respectively. Glycogen supplementation significantly increased PYGM and LDH activities by approximately 2-fold in dark-cutting samples after 24 h of incubation (Figure 2D,E, *p* < 0.05). PYGM activity showed a numerical increase in dark-cutting beef compared to normal beef control (both without any added glycogen) at both 1 and 24 h of incubation (Figure 2D and Figure S1A). In contrast, LDH activity was significantly lower at 1 h of incubation (Figure 2E and Figure S1B). These results suggest that substrate levels (glycogen content), rather than enzyme abundances, predominately influence postmortem pH decline in dark-cutting beef muscle.

To assess the impact of glycogen supplementation on postmortem metabolism pathways, we further examined activities of adenosine monophosphate activated protein kinase (AMPK) and its phosphorylated form (pAMPK). Glycogen supplementation significantly decreased the AMPK activity after 1 h of incubation (*p* < 0.05, Figure S1C). In contrast, pAMPK activity remained unchanged compared with control groups (without added glycogen) throughout 1 and 24 h of incubation (Figure 2H and Figure S1D). Interestingly, untreated dark-cutting samples exhibited numerically greater AMPK activity (not statistically significant) than normal-pH beef after 1 h of incubation (Figure S1C). These findings suggest that the pH decline observed following glycogen supplementation may be influenced by increased substrate availability and/or via substrate induced activation of glycogen metabolizing enzymes, with minimal impact on overall energy balance.

### Inhibiting Mitochondrial Oxidative Metabolism Following in Vitro Glycogen Supplementation Promotes Greater pH Decline in Post Mortem Dark-Cutting Beef Treatments

**Figure 3:**
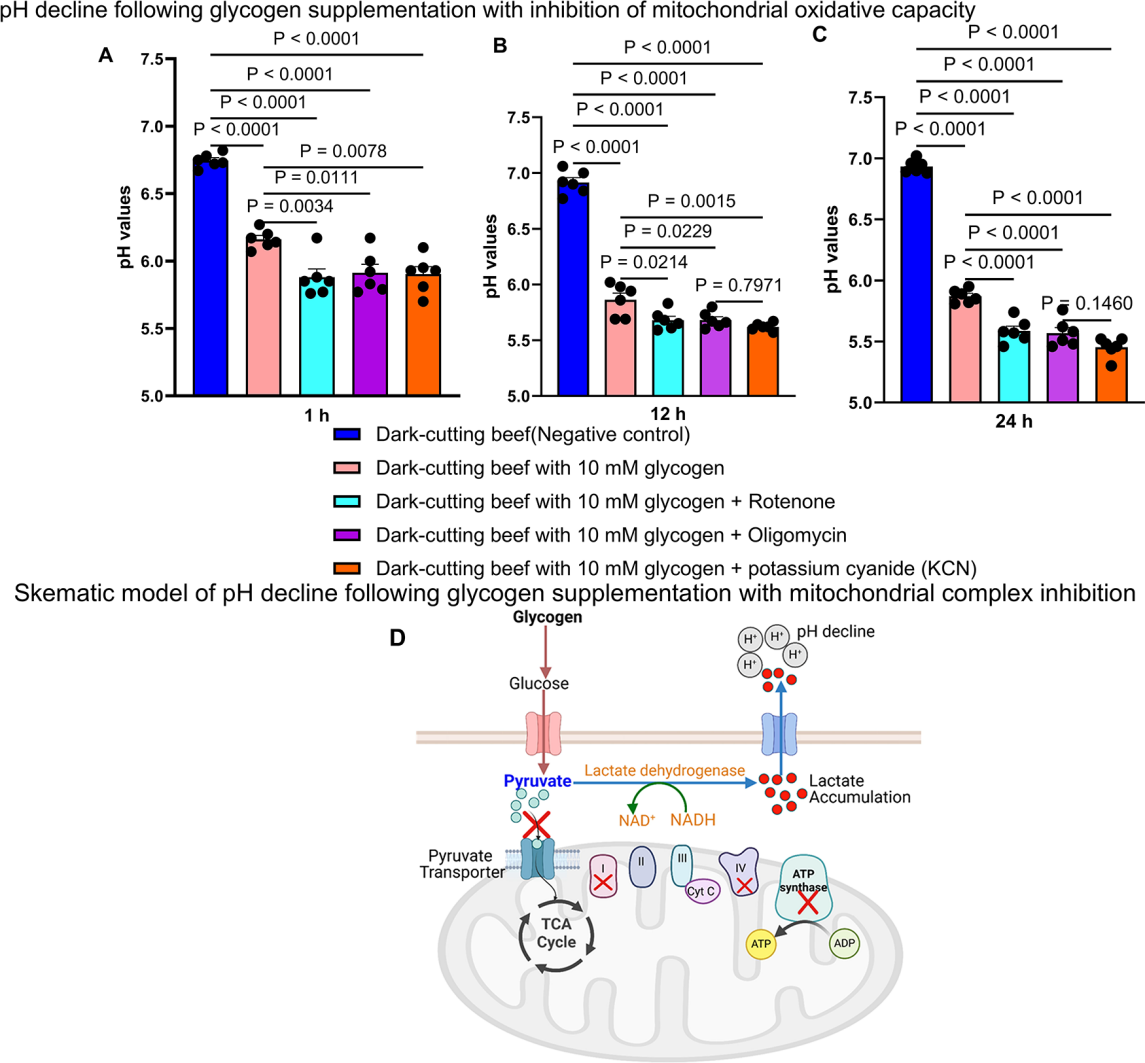
Inhibiting mitochondrial oxidative capacity coupled with glycogen supplementation promotes greater pH decline in dark-cutting beef treatments. (A–D) Summary of changes in muscle pH levels in response to 10 mM glycogen supplementation coupled with mitochondrial inhibition at complexes I (2 μM rotenone), IV (1 mM potassium cyanide), and V (2 μM oligomycin, *n* = 6 per group. ANOVA, *p* < 0.001). (D) Schematic representation of a model depicting pH decline following glycogen supplementation in dark-cutting beef muscles. Error bars represent ± standard error of mean (SEM) with *p* values; each dot in a bar graph represents a biological replicate.

Using glycogen supplementation and inhibition of mitochondrial oxidative capacity at complexes I, IV, and V, pH measurements showed an increase (*p* < 0.05) in pH decline (∼15%, after 1 h, Figure 3A). Interestingly, by 24 h of incubation, suppression of mitochondrial oxidative capacity at complexes (I, IV, and V) increased pH decline (*p* < 0.05), registering similar pH values to intact postmortem normal beef muscles (pH values = 5.6; Figure 3C). These results suggest that inhibition of mitochondria function might, in part, limit the transport of glycolytic end-product pyruvate into the mitochondria for oxidation, favoring subsequent accumulation of lactate (Figure 3D).

### Glycogen Supplementation Alters Myoglobin Redox Stability in Dark-Cutting Muscles

**Figure 4:**
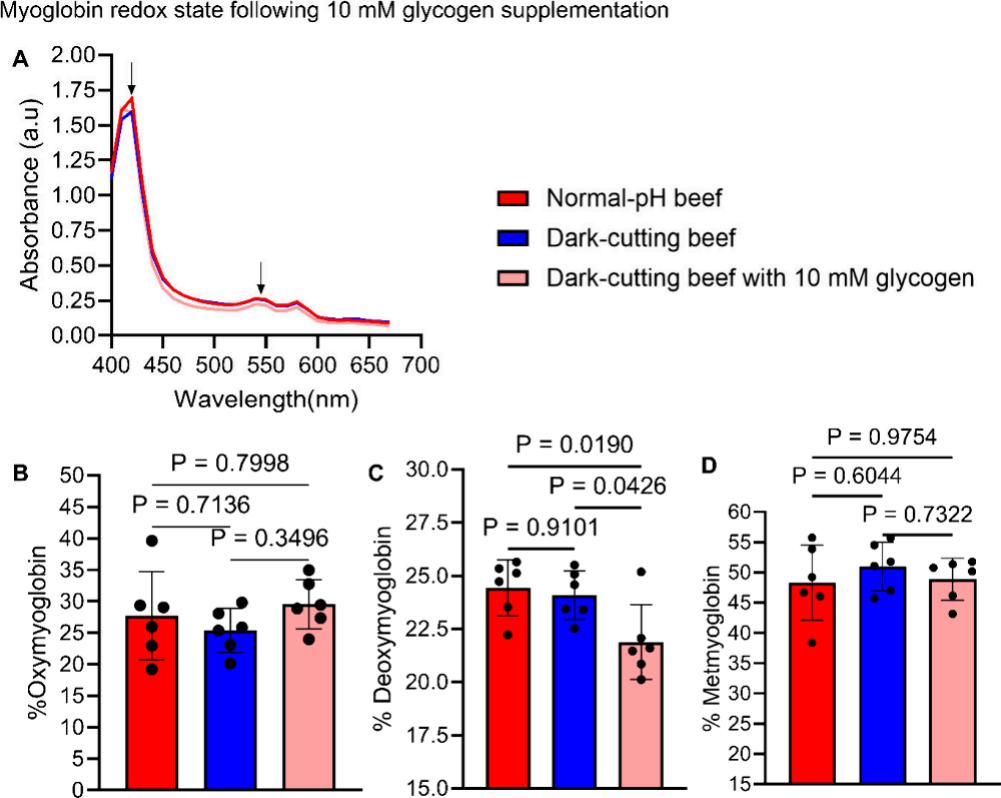
Effect of glycogen supplementation on myoglobin redox state. (A) Comparison of absorbance spectra of myoglobin in normal beef (red) versus dark-cutting beef with or without 10 mM glycogen supplementation (light red and blue, respectively). (B–D) Summary of changes in the oxymyoglobin (B), deoxymyoglobin (C), and metmyoglobin (D) levels after 24 h of incubation with or without 10 mm glycogen (*n* = 6 per group, ANOVA, p < 0.05). Error bars represent ± standard error of mean (SEM) with *p* values; each dot in a bar graph represents a biological replicate.

The observed effects of in vitro glycogen supplementation on pH decline (Figure 2B–D) influenced composition of myoglobin redox state (Figure 4). Glycogen supplementation significantly reduced deoxymyoglobin content after 24 h of incubation compared with untreated control groups (Figure 4C). Although oxymyoglobin levels increased numerically (*p* > 0.05) with glycogen supplementation, metmyoglobin content remained unaffected (Figure 4B,D). The reduction in deoxymyoglobin may be linked to increased pH decline postglycogen supplementation (Figure 2B–D), implying potential changes in color profiles in dark-cutting beef due to more myoglobin oxygenation.

### Glycogen Supplementation Restores pH Decline in Dark-Cutting Beef Treatments by Reverting the Metabolome toward a Typical Postmortem Muscle State

**Figure 5:**
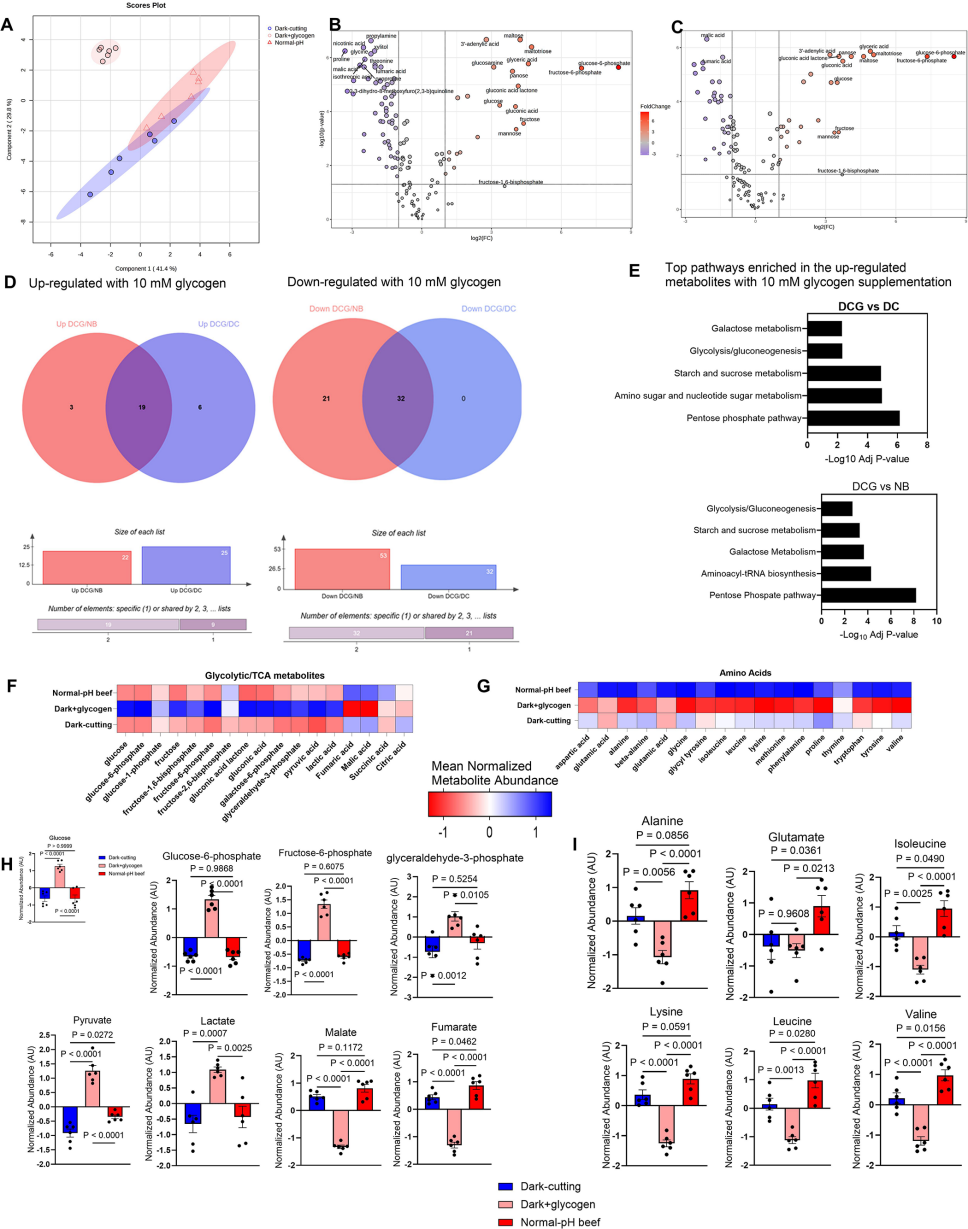
Glycogen supplementation induces a metabolic shift toward typical postmortem muscle state. (A) Partial least-squares discrimination (PLS-DA) analysis of metabolite profiles in normal beef (red) versus dark-cutting beef with or without 10 mM glycogen supplementation (light red and blue, respectively). (B,C) Volcano plots visualization of differentially abundant metabolites in treated dark-cutting beef versus normal beef (B) and untreated dark-cutting beef (C). Metabolites with a false discovery rate (FDR)-adjusted *p* value <0.05 and a Log2-fold change (FC) >2 or less than −2 were considered differentially abundant. (D) Venn diagrams of differentially abundant metabolites up-and down-regulated in (B) and (C). (E) Representative metabolites enriched in upregulated pathways in glycogen treated dark-cutting vs untreated (upper panel) and glycogen treated dark-cutting vs normal beef (lower panel) after 24 h of incubation, indicating shifts toward biosynthetic pathways. (F,G) Heat maps illustrating normalized metabolite abundance for glycolytic and tricarboxylic acid cycle-related metabolites (F) and amino acid metabolism (G), following with glycogen at 24 h of incubation. (H) Boxplots displaying highlighted glycolytic and TCA metabolites altered with glycogen supplementation. (I) Boxplots demonstrating highlighted amino acid metabolites affected by glycogen supplementation after 24 h of incubation. (*n* = 6 per group, ANOVA, *p* < 0.05). Error bars represent ± standard error of the mean (SEM) with *p* values; each dot in a bar graph represents a biological replication.

To gain further insight into how glycogen might play a direct role in regulating muscle pH decline in postmortem dark-cutting beef, we conducted nontargeted metabolomics profiling via gas chromatography–mass spectrometry (GC-MS/MS). A total of 136 metabolite features were identified across sample groups after 24 h of incubation. Principle component analysis (PCA) revealed distinct clustering by treatments after 24 h of incubation (Figure 5A), with glycogen supplemented treatments showing separate clustering from both dark-cutting and normal beef control groups (with no added glycogen). Pairwise comparison of metabolic profiles between glycogen supplemented dark-cutting and untreated dark-cutting control samples revealed 25 overabundant and 31 less abundant metabolites (FDR <0.05; >±2-fold change, Figure 5C,D), while comparison with normal beef control showed 22 overabundant and 55 less abundant metabolites (FDR <0.05; >±2-fold change, Figure 5B–D). Furthermore, pathway analysis indicated a greater enrichment in energy biosynthetic and storage pathways, specifically, the pentose phosphate pathway (Figure 5E).

Examination of the specific differentially abundant metabolites revealed a strikingly greater abundance of glycolytic metabolites in glycogen supplemented dark-cutting samples compared to control groups after 24 h of incubation (FDR <0.05; Figure 5,H). This increase in glycolytic metabolite abundance coincided with reduced levels of tricarboxylic acid cycle, amino acids, and nucleotide metabolites in glycogen supplemented dark-cutting samples (FDR <0.05; Figure 5G,I, and Figure S2). Taken together, our metabolomics data suggest that glycogen plays a crucial role in postmortem pH decline, and adequate glycogen supplementation to dark-cutting muscles could trigger a metabolic shift capable of restoring typical postmortem metabolic programs and pH decline.

### Metabolomics Profiling Reveals Time-Dependent Effects of Glycogen Supplementation on pH Decline in Dark-Cutting Treatments

To explore glycogen’s time-dependent impact on pH decline, we analyzed metabolite profiles of glycogen supplemented dark-cutting samples at 1 and 24 h of incubation (Figure S3). PCA revealed distinct metabolite clusters of metabolites at both time points, with component 1 explaining 88.4% of the total variance (Figure S3A). Among 105 differentially abundant metabolites, 66 were overabundant and 39 were less abundant after 24 h of incubation (Figure S3B). Pathway analysis indicated enrichment of metabolites related to pyruvate, citric acid cycle, and starch metabolism (Figure S3C), while metabolites associated with the pentose phosphate pathways were less enriched after 24 h (Figure S3D), suggesting increased availability of glycolysis intermediates and reduced biosynthetic pathways (Figure S3E,F).

### Concurrent Inhibition of Mitochondria Oxidative Capacity with Glycogen Supplementation Highlight Electron Transport Chain Complex-Specific Impacts on Metabolic Pathways and pH Regulation

**Figure 6:**
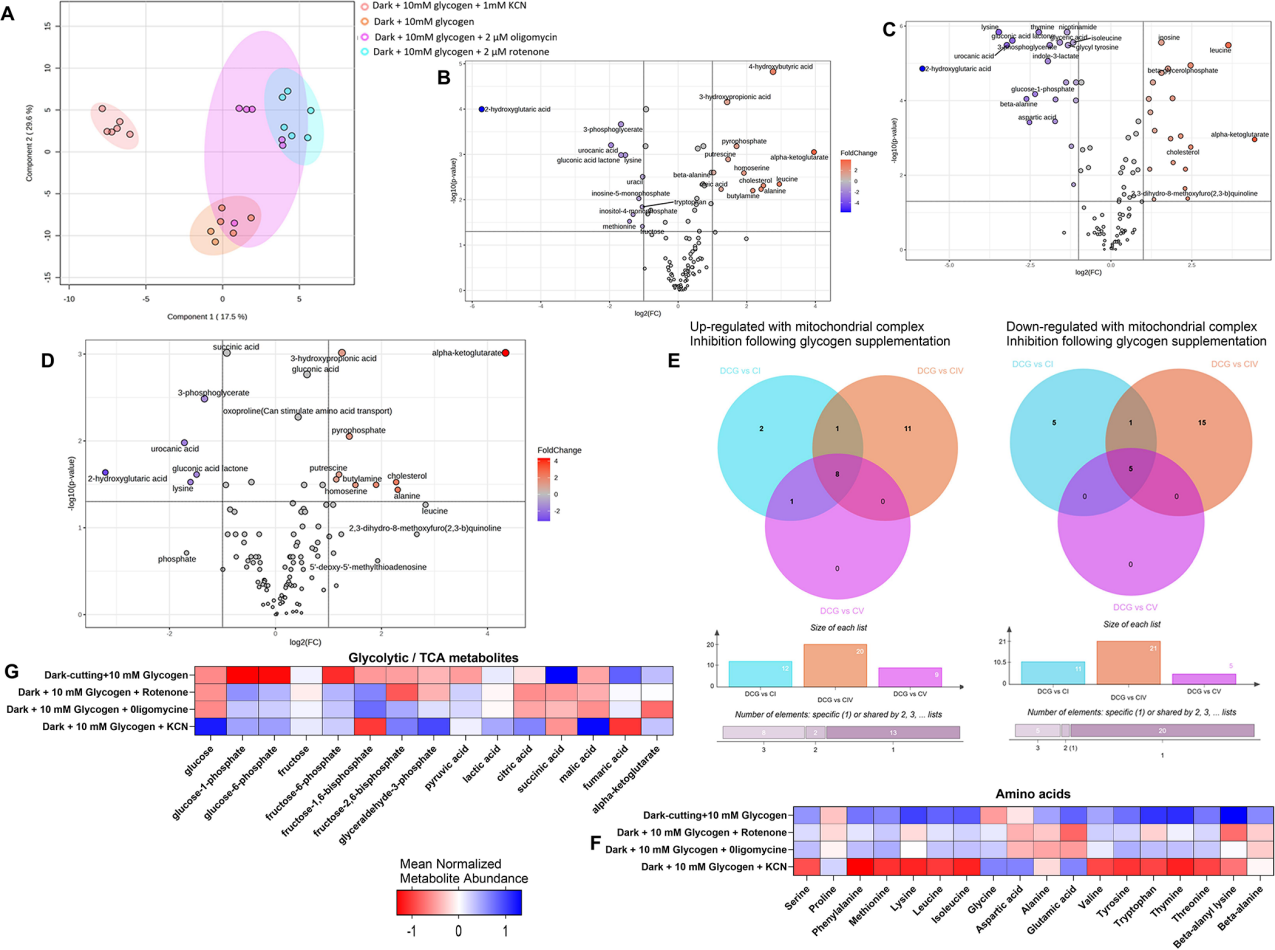
Metabolite expression changes in dark-cutting beef treated with 10 mM glycogen and mitochondrial inhibitions at complexes I, IV, and V. (A) Partial least-squares discrimination (PLS-DA) plot. (B,C) Volcano plot visualizing the differentially abundant metabolites in glycogen treated dark-cutting group coupled with inhibition of mitochondrial oxidative metabolism at complexes I (2 μM rotenone (B)), IV (1 mM potassium cyanide (C)), and V (2 μM oligomycin (D)). Metabolites with a false discovery rate (FDR)-adjusted *p* value <0.05 and a Log2-fold change (FC) >2 or less than −2 were considered differentially abundant. The Log2-fold change in metabolite abundance was plotted against the Log10 (FDR)-adjusted *p* value for each metabolite. (E) Venn diagrams represent up-regulated (left panel) and down-regulated (right panel) metabolites following mitochondrial inhibition at complex I (light blue), IV (orange), and V (purple). (F,G) Heat map representation of specific metabolite sets that show enrichment in glycolytic and tricarboxylic acid cycle (F) and amino acid (G) metabolism following glycogen supplementation in dark-cutting beef at 24 h of incubation.

We further examined the combined impact of mitochondrial inhibition at complexes I, IV, and V with glycogen supplementation on the metabolites profiles. Principal component analysis revealed a clear separation between the glycogen-supplemented group and mitochondrial-inhibited groups, with less distinction among the inhibited mitochondrial complexes (Figure 6A). Overall, fewer metabolite changes (Figure 6B–D) were observed, and distinct metabolic pathways were enriched following the inhibition of mitochondrial oxidative capacity at specific complexes (Figure 6F,G). Across all inhibited mitochondrial complexes, differentially abundant metabolites were primarily related to glycolytic, TCA (Figure 6F), and amino acid pathways (Figure 6G). Specifically, inhibition of mitochondrial oxidative capacity at complex IV, along with glycogen supplementation, led to a greater number of uniquely differentially abundant metabolites (Figure 6E), predominantly associated with glycolytic metabolism, and reduced levels of amino acid metabolites (Figure 6F,G). These findings suggest that glycogen supplementation, combined with inhibition of mitochondrial function at complex IV, induces a metabolic reprograming capable of driving pH decline in dark-cutting beef muscles.

## Discussion

Altered glycogen metabolism is a well-characterized hallmark of muscle darkening in beef, marked by reduced carbohydrate metabolism and increased amino and fatty acid metabolism.^10,36−39^ This metabolic alteration leads to diminished glycolytic flux, resulting in reduced lactic acid formation and higher postmortem muscle pH values.^6−12^ Despite this understanding, the precise mechanism and cellular targets driving these changes remain unclear. In this study, we explore the metabolic role of glycogen in beef muscle darkening and pH decline. Our findings suggest a model where inherent substrate inhibition mechanisms in dark-cutting beef muscles result in deficiencies in substrate selection, triggering a metabolic reprogramming characterized by limited substrate utilization flexibility postmortem.

Our findings demonstrate that glycogen levels, rather than enzyme abundances, may represent the principal limiting factor in the pH decline of dark-cutting beef muscles. Consistent with this observation, elevating glycogen levels in dark-cutting beef muscles initiated a metabolic shift capable of restoring typical postmortem pH decline and metabolic patterns (Figures 2 and 5). This restoration is attributed to glycogen’s role in modulating postmortem glycolysis.^40−42^ Increased glycogen levels likely augment the muscles’ capacity for net glycogen breakdown, resulting in a more pronounced pH decline after 24 h of incubation (Figure 2B–D). This accelerated pH decline is partly driven by substrate-dependent activation of enzymes involved in glycogen breakdown and lactic acid formation. Notably, glycogen supplementation induced approximately a 2-fold increase in activities of glycogen phosphorylase and lactate dehydrogenase in dark-cutting samples after 24 h of incubation (Figure 2D, 2E, Figure S1A,B). This observation aligns with previous reports that glycogen phosphorylase, an enzyme catalyzing the rate-limiting step in glycogen breakdown to produce glucose-1-phosphate,^43−45^ is activated by increased glycogen concentration through a substrate positive feedback loop.^46^ Thus, the lower abundance of glycogen breakdown and lactate formation enzymes documented in dark-cutting phenotypes^6,8,47^ may reflect an energy conservation mechanism mediated by substrate availability.

Elevated rates of pH decline, a hallmark of normal beef, stem from heightened glycolytic flux, coupled with a compensatory reduction in tricarboxylic acid cycle and amino acid metabolites.^12,48^ Metabolomic profiling revealed a similar metabolic shift induced by glycogen supplementation in dark-cutting samples, characterized by elevated levels of carbohydrate metabolites (e.g., glucose, glucose-6-phosphate, fructose, fructose-6-phosphate, glyceraldehyde-3-phosphate, pyruvate, and lactate; Figure 5F,H, and Figure S2), and reducing tricarboxylic cycle and amino acid metabolites (Figure 5G,I). This suggests that glycogen supplementation boosts glycolysis in vitro in dark-cutting beef, augmenting glycolytic flux and lactate production by activating glycogen breakdown (PGYM) and lactate formation (LDH) enzymes. The reduced activity in the tricarboxylic acid cycle and amino acid metabolism may, in part, result from a redirection in metabolic flux toward biosynthetic pathways such as the pentose phosphate pathway (Figure 5E). Thus, this metabolic reprogramming reflects an adaptive response to altered substrate availability and utilization flexibility, highlighting the intricate alterations in cellular metabolism in dark-cutting beef following glycogen supplementation, impacting both energy homeostasis and biosynthetic processes.

While muscle pH decline typically follows glycolysis and lactic acid accumulation, postmortem metabolism also engages adenylate kinases mediated energy pathways.^20^ AMPK, a pivotal regulator of energy homeostasis, responds to metabolic cues impacting ATP synthesis and consumption.^49−51^ We hypothesized that dark-cutting beef muscles might activate adenylate kinase networks to modulate energy balance. However, our assessment revealed only a marginal increase in AMPK activity in dark-cutting compared to normal beef control treatments (Figure S1C), potentially reflecting variances in substrate sensitivity and the prevalence of oxidative fibers in dark-cutting muscles.^12,52^ Interestingly, glycogen supplementation reduced AMPK activity in dark-cutting samples within 1 h (Figure S1C), likely attributable to glycogen loading’s inhibition of AMPK activation via binding at the β subunit of the glycogen binding domain^53−55^ or elevated ATP levels stemming from augmented glycolytic flux (Figure 5D), thereby impeding AMPK activity.^51,53,54^ These findings suggest that in dark-cutting beef muscles, fuel-driven mechanisms, rather than energy status per se, may regulate AMPK signaling, contradicting the idea that preslaughter energy fluctuations activate adenylate kinase networks in dark-cutting beef.

The intricate interplay between glycolysis and mitochondrial function regulates substrate metabolism, a phenomenon critical for understanding postmortem muscle dynamics. Elevated oxidative metabolism observed in dark-cutting beef postmortem^13,56,57^ potentially counterbalances muscle acidification by enhancing the utilization of glycolytic end products in mitochondrial oxidative pathways. In our study, concurrent inhibition of mitochondrial oxidative capacity and glycogen supplementation in dark-cutting treatments accentuated the pH decline (Figure 3A–C). This phenomenon is attributed to (i) the accumulation of glycolytic metabolites such as pyruvate (Figure 5F,H), which can undergo conversion to lactate,^14,16,18,58−60^ and (ii) reduced pyruvate translocation into the mitochondrial for oxidation. Although direct measurements of specific mitochondrial pyruvate carriers (MPC1 and MPC2), pyruvate dehydrogenase, and pyruvate carboxylase protein activities and abundance were not conducted, our findings align with previous studies showing that lactate dehydrogenase sustains glycolytic flux under limited mitochondrial oxidation.^61,62^ Moreover, impaired mitochondrial oxidative capacity correlated with decreased alanine levels (Figure 6F and Figure S2), suggesting diminished pyruvate transport into the mitochondria^63,64^ and a plausible mechanism for heightened pH decline via lactate dehydrogenase enzyme catalysis (Figure 3D). Thus, our results highlight the pivotal role of enhanced mitochondria oxidative metabolism in modulating substrate utilization flexibility within postmortem dark-cutting muscles, offering insights into potential avenues for enhancing meat quality.

While the impact of molecular associations between glycogen and myoglobin on meat color remains unexplored, our data unveil a potential link between glycogen levels and myoglobin redox stability in dark-cutting beef (Figure 4). Notably, a substantial decrease in deoxymyoglobin was observed following 24 h of glycogen supplementation (Figure 4C), possibly driven by (i) glycogen-induced pH decline (Figure 2C), (ii) heightened glycolytic flux (Figure 5F and Figure S2), and (iii) decrease in mitochondria activity due to increased pH decline leading to less mitochondrial oxygen consumption and myoglobin deoxygenation. These findings align with prior studies demonstrating that glucose, a byproduct of glycogen breakdown, accelerates the conversion of met-heme to ferryl-heme when bound within myoglobin xenon cavities,^65^ and the regulatory influence of glycolytic end products, pyruvate and lactate, on oxygen availability under metabolically activated conditions.^66^ However, further investigations are warranted to unravel the intricate mechanisms dictating the efficacy of glycogen supplementation in vivo and devise refined strategies for enhancing the meat color profiles of dark-cutting beef.

In conclusion, our study highlights the pivotal role of glycogen in orchestrating postmortem metabolic dynamics and muscle pH regulation in dark-cutting beef. Notably, glycogen supplementation, particularly at 10 mM, exerts a profound influence on pH decline by stimulating key enzymes involved in glycogen catabolism and lactate production, thereby reshaping metabolic pathways toward a typical postmortem muscle state. These changes are marked by elevated glycolytic metabolites and diminished tricarboxylic acid cycle (TCA) intermediates and amino acids. Furthermore, concurrent supplementation of glycogen with inhibition of mitochondrial oxidative capacity, specifically at complex IV, synergistically augments a greater pH decline. These findings collectively support previous studies that reported limited glycogen deposition and less active glycogen breakdown enzymes leading to aberrant postmortem muscle pH decline and associated quality issues.

## Supporting Information Available

Figures S1−S3 (PDF)
