# Supplementary material for: Glycogen Supplementation in Vitro Promotes pH Decline in Dark-Cutting Beef by Reverting Muscle’s Metabolome toward a Normal Postmortem Muscle State
Source: J Agric Food Chem. 2024 Nov 4;72(45):25275–85. doi: 10.1021/acs.jafc.4c06490 (PMC11565789; doi:10.1021/acs.jafc.4c06490)
Supplement: Supplementary file 1 — jf4c06490_si_001.pdf [file jf4c06490_si_001.pdf]

# Supplemental Information

# Glycogen supplementation in-vitro promotes pH decline in dark-cutting beef by reverting muscle's metabolome towards normal postmortem muscle state

Frank Kiyimba[a], Steven D. Hartson[b], Gretchen G. Mafi[a], and Ranjith Ramanathan[a*]

[*]Corresponding author: Email: Ranjith.Ramanathan @okstate.edu

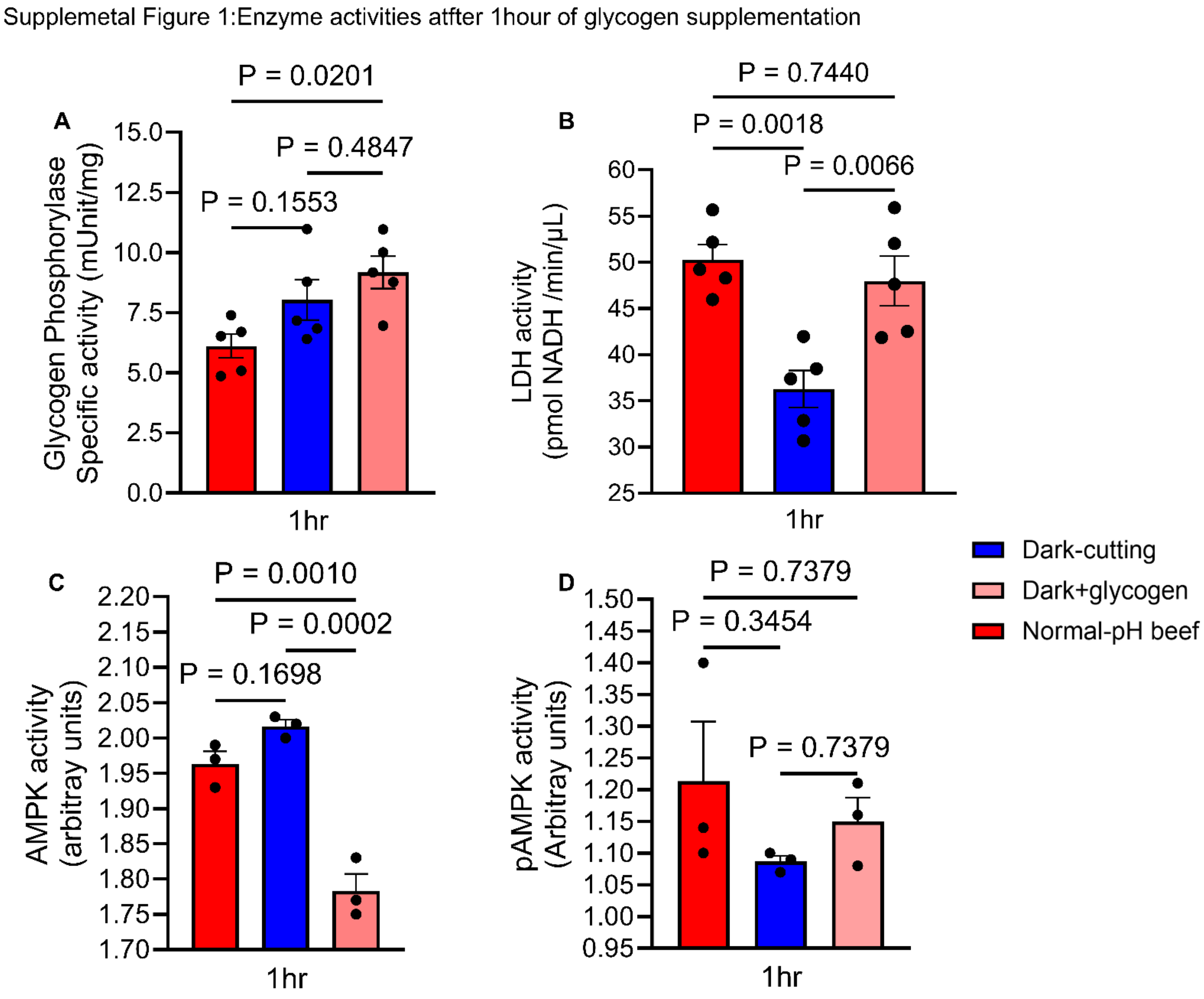


**Supplemental Figure 1**: Enzyme activity alterations post-glycogen supplementation after 1 hour of incubation. A: Glycogen phosphorylase activity (n = 5 per group). B: Lactate dehydrogenase activity (n = 5 per group). C: AMPK activity (n = 3 per group) and D: pAMPK activity (n = 3 per group). Error bars represent ± standard error of mean (SEM), with exact p-values provided; each dot in a bar graph represents a biological replicate.

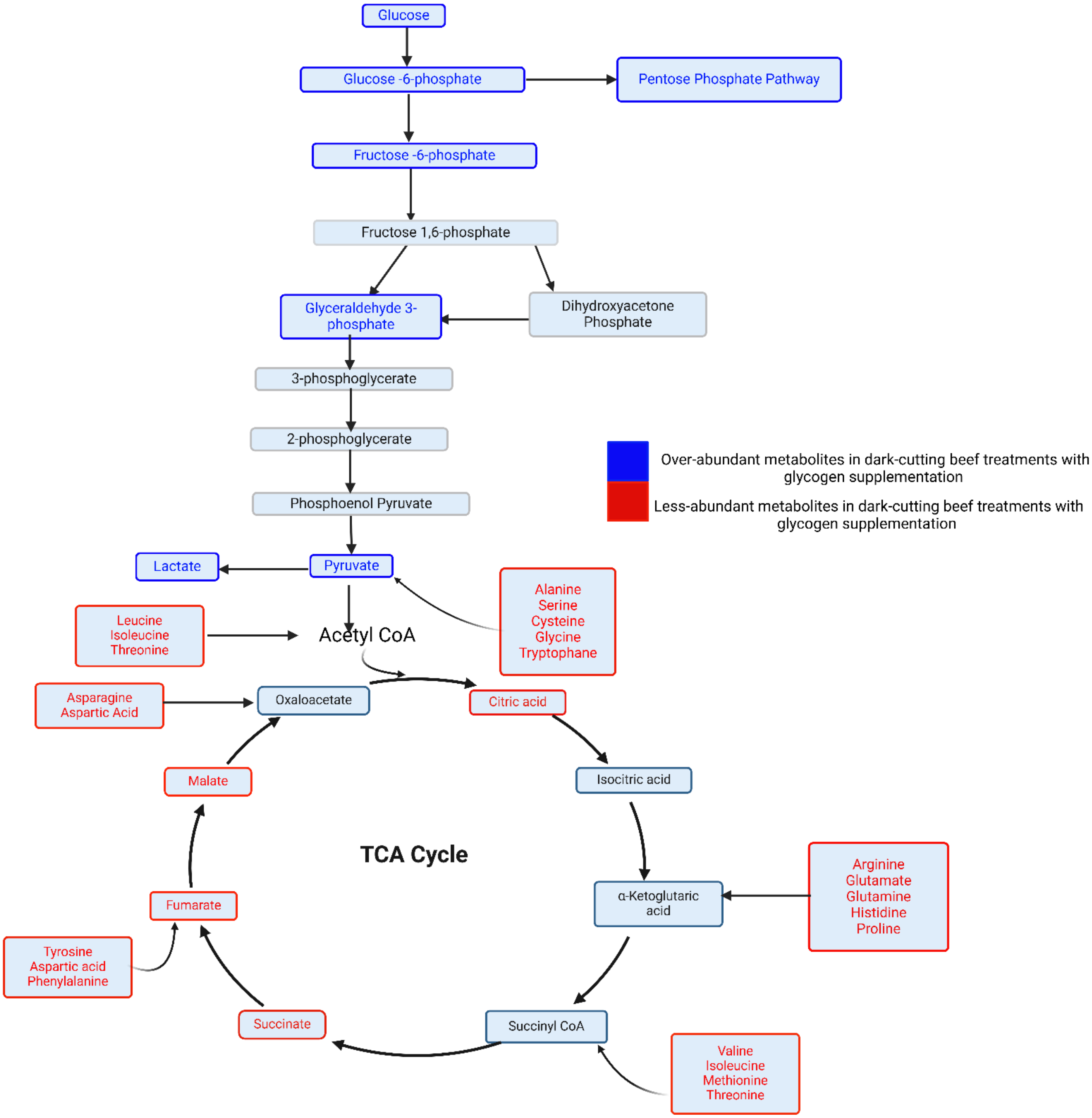


**Supplemental Figure 2:** Summary diagram illustrating the impact of glycogen supplementation on glycolytic and tri carboxylic acid metabolites in dark-cutting compared to untreated dark-cutting control groups. Illustration was constructed using Biorender (https://app.biorender.com).

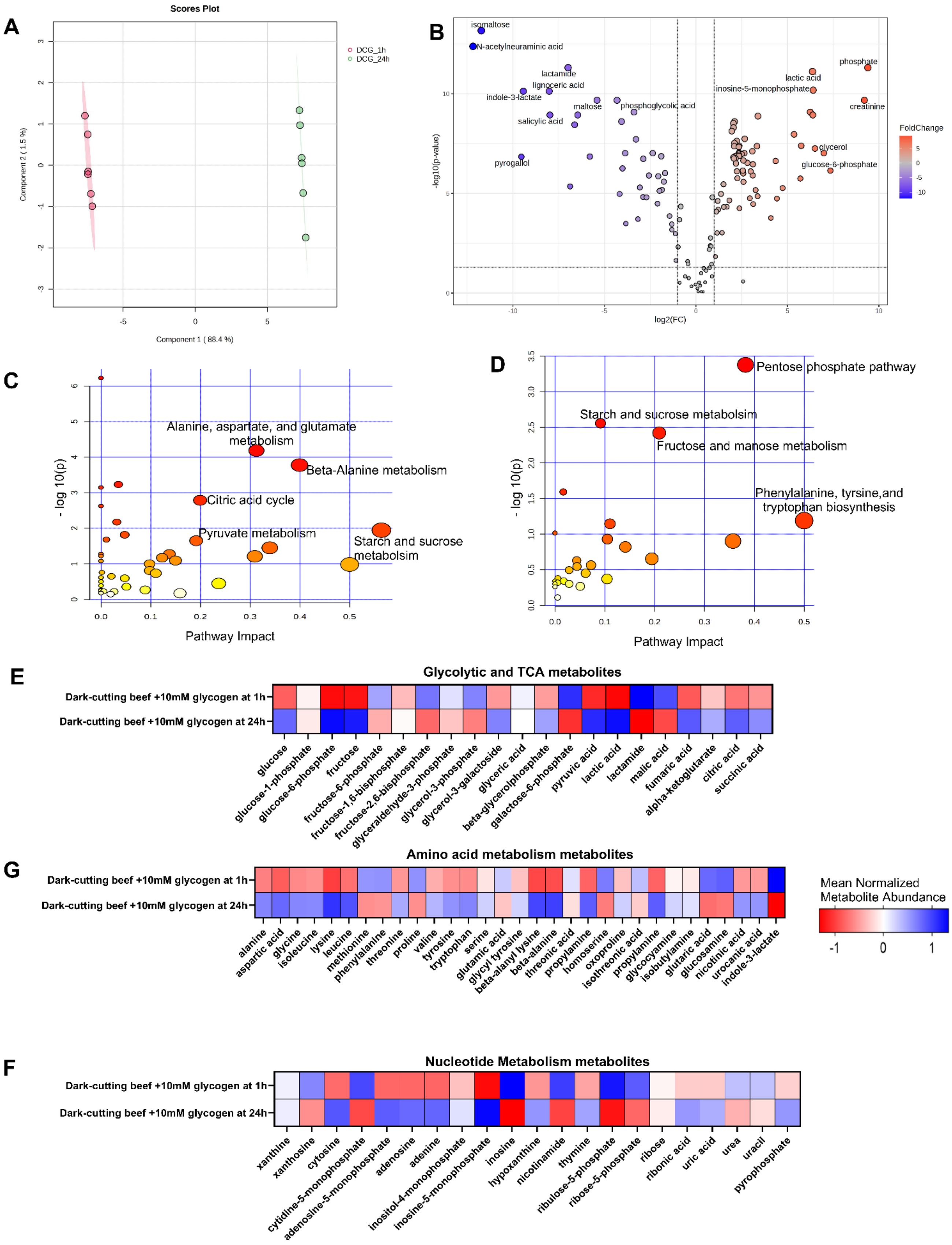
A
Scores Plot
DCG_1h
DCG_24h
Component 1 ( 88.4 %)
Component 2 ( 1.5 %)
B
isomaltose
N-acetylneuraminic acid
lactamide
lignoceric acid
indole-3-lactate
maltose
phosphoglycolic acid
salicylic acid
pyrogallol
phosphate
lactic acid
inosine-5-monophosphate
creatinine
glycerol
glucose-6-phosphate
FoldChange
log2(FC)
-log10(p-value)
C
Alanine, aspartate, and glutamate metabolism
Beta-Alanine metabolism
Citric acid cycle
Pyruvate metabolism
Starch and sucrose metabolsim
- log 10(p)
Pathway Impact
D
Pentose phosphate pathway
Starch and sucrose metabolsim
Fructose and manose metabolism
Phenylalanine, tyrsine,and tryptophan biosynthesis
- log 10(p)
Pathway Impact
E
Glycolytic and TCA metabolites
Dark-cutting beef +10mM glycogen at 1h
Dark-cutting beef +10mM glycogen at 24h
glucose
glucose-1-phosphate
glucose-6-phosphate
fructose
fructose-6-phosphate
fructose-1,6-bisphosphate
fructose-2,6-bisphosphate
glyceraldehyde-3-phosphate
glycerol-3-phosphate
glycerol-3-galactoside
glyceric acid
beta-glycerolphosphate
galactose-6-phosphate
pyruvic acid
lactic acid
lactamide
malic acid
fumaric acid
alpha-ketoglutarate
citric acid
succinic acid
G
Amino acid metabolism metabolites
Dark-cutting beef +10mM glycogen at 1h
Dark-cutting beef +10mM glycogen at 24h
alanine
aspartic acid
glycine
isoleucine
lysine
leucine
methionine
phenylalanine
threonine
proline
valine
tyrosine
tryptophan
serine
glutamic acid
glycyl tyrosine
beta-alanyl lysine
beta-alanine
threonic acid
propylamine
homoserine
oxoproline
isothreonic acid
propylamine
glycocyamine
isobutylamine
glutaric acid
glucosamine
nicotinic acid
urocanic acid
indole-3-lactate
Mean Normalized
Metabolite Abundance
-1
0
1
F
Nucleotide Metabolism metabolites
Dark-cutting beef +10mM glycogen at 1h
Dark-cutting beef +10mM glycogen at 24h
xanthine
xanthosine
cytosine
cytidine-5-monophosphate
adenosine-5-monophosphate
adenosine
adenine
inositol-4-monophosphate
inosine-5-monophosphate
inosine
hypoxanthine
nicotinamide
thymine
ribulose-5-phosphate
ribose-5-phosphate
ribose
ribonic acid
uric acid
urea
uracil
pyrophosphate

**Supplemental Figure 3:** Metabolite expression changes in dark-cutting beef treated with 10 mM glycogen after 1 and 24 hours of incubation. A: Partial least square discrimination (PLS-DA) plot. B: Volcano plot visualizing the differentially abundant metabolites in treated dark-cutting beef. Metabolites with a false discovery rate (FDR)-adjusted P value < 0.05 and a Log2fold change (FC) > 2 or less than -2 were considered differentially abundant. The Log2 fold change in metabolite abundance was plotted against the- Log10 (FDR)-adjusted p-value for each metabolite. C-D: Pathway enrichment analysis of differentially abundant metabolites, highlighting over abundant (C) and less abundant (D) with glycogen supplementation after 24 hours of incubation. E-F: Heat map representation of specific metabolite sets showing enrichment in glycolytic and tri carboxylic acid cycle (E), Amino acid (F), and nucleotide metabolism (G) following with glycogen supplementation in dark-cutting beef at 1 versus 24 hours of incubation
